# Low-Dose CT Quality Assurance at Scale: Automated Detection of Overscanning, Underscanning, and Image Noise

**DOI:** 10.3390/life16010152

**Published:** 2026-01-16

**Authors:** Patrick Wienholt, Alexander Hermans, Robert Siepmann, Christiane Kuhl, Daniel Pinto dos Santos, Sven Nebelung, Daniel Truhn

**Affiliations:** 1 Lab for Artificial Intelligence in Medicine, Department of Diagnostic and Interventional Radiology, University Hospital RWTH Aachen, 52074 Aachen, Germany; 2 Department of Diagnostic and Interventional Radiology, University Hospital RWTH Aachen, 52074 Aachen, Germany; 3 Visual Computing Institute, RWTH Aachen University, 52074 Aachen, Germany; 4 Department of Diagnostic and Interventional Radiology, University Medical Center of Johannes Gutenberg-University Mainz, 55131 Mainz, Germany

**Keywords:** low-dose computed tomography (LDCT), lung cancer screening, automated quality assurance, scan coverage, overscanning, underscanning, image noise, radiation dose optimization, National Lung Screening Trial (NLST)

## Abstract

Automated quality assurance is essential for low-dose computed tomography (LDCT) lung screening, yet manual checks strain clinical workflows. We present a fully automated artificial intelligence tool that quantifies scan coverage and image noise in LDCT without user input. Lungs and the aorta are segmented to measure cranial/caudal over- and underscanning, and noise is computed as the standard deviation of Hounsfield units (HUs) within descending aortic blood, normalized to a 1 ${\mathrm{mm}}^{3}$ voxel. Performance was verified in a reader study of 98 LDCT scans from the National Lung Screening Trial (NLST), and then applied to 38,834 NLST scans reconstructed with a standard kernel. In the reader study, lung masks were rated ≥“Nearly Perfect” in 90.8% and aorta-blood masks in 96.9% of cases. Across 38,834 scans, mean overscanning distances were 31.21 mm caudally and 14.54 mm cranially; underscanning occurred in 4.36% (caudal) and 0.89% (cranial). The tool enables objective, large-scale monitoring of LDCT quality—reducing routine manual workload through exception-based human oversight, flagging protocol deviations, and supporting cross-center benchmarking—and may facilitate dose optimization by reducing systematic over- and underscanning.

## 1. Introduction

With more than 2.4 million new cases and over 1.8 million deaths in 2022 alone, lung cancer is the most common cancer and the leading cause of cancer-related deaths worldwide [1]. The 5-year survival rate in Europe is estimated to be approximately 10.9% [2]. However, early-stage detection (stage Ia) can significantly improve survival, with rates exceeding 70% when appropriate treatment is administered [3]. Screening serves as an important strategy for facilitating early detection and timely intervention. In parallel, artificial intelligence (AI) methods such as machine learning and deep learning are increasingly used across medicine to support and automate diagnostic workflows [4].

The effectiveness of screening is demonstrated by the National Lung Screening Trial (NLST) [5], which showed that low-dose helical computed tomography (LDCT) can lead to a 20% reduction in mortality compared to radiography-based screening [6]. LDCT screening also reduced the overall death rate by 6.7% [5]. These findings have driven several countries, such as Croatia, South Korea, and the United States, to introduce lung cancer screening programs, while others, including Germany, the United Kingdom, and Australia, are in the process of implementing them [7]. International LDCT screening is commonly embedded in QA (Quality Assurance) frameworks that combine standardized reporting with structured audit and benchmarking. In the US, Lung-RADS supports consistent reporting and outcome monitoring [8]. In England, NHS England publishes programme-level QA standards for LDCT screening delivery [9]. ESR/ERS guidance similarly emphasizes comprehensive, quality-assured longitudinal screening programmes [10]. In line with these standards, our tool provides automated acquisition-level QA metrics (scan-range coverage and in-vivo noise) to support monitoring, benchmarking, and exception-based human review.

Kim et al. [11] proposed a deep learning–based overscan decision algorithm that segments the thyroid cartilage and kidneys instead of the lungs to define the superior and inferior scan limits. Because these landmarks vary in their position relative to the lung apices and bases, their distance to the true cranial and caudal lung borders differs between patients.

Variations in procedures across institutions conducting LDCT scans under different conditions result in inconsistencies in scan quality [12]. Early identification and correction of these variations are crucial. However, given the already intensive clinical workloads, manual quality assessment would impose an additional burden, potentially reducing the number of patients treated and hindering effective care.

To address this issue, we have developed a fully automated AI-based tool that assesses the quality of LDCT scans with no additional user interaction in routine cases and provides outputs intended to support radiologists and technologists in QA workflows. Our tool evaluates two key parameters: overscanning, which indicates unnecessary tissue exposure, and image noise, which reflects the radiation dose [13]. Additionally, the tool can generate a PDF report summarizing key parameters for quick review and archiving, and it can be integrated into other software solutions for continuous background monitoring or retrospective analysis.

**Figure 1:**
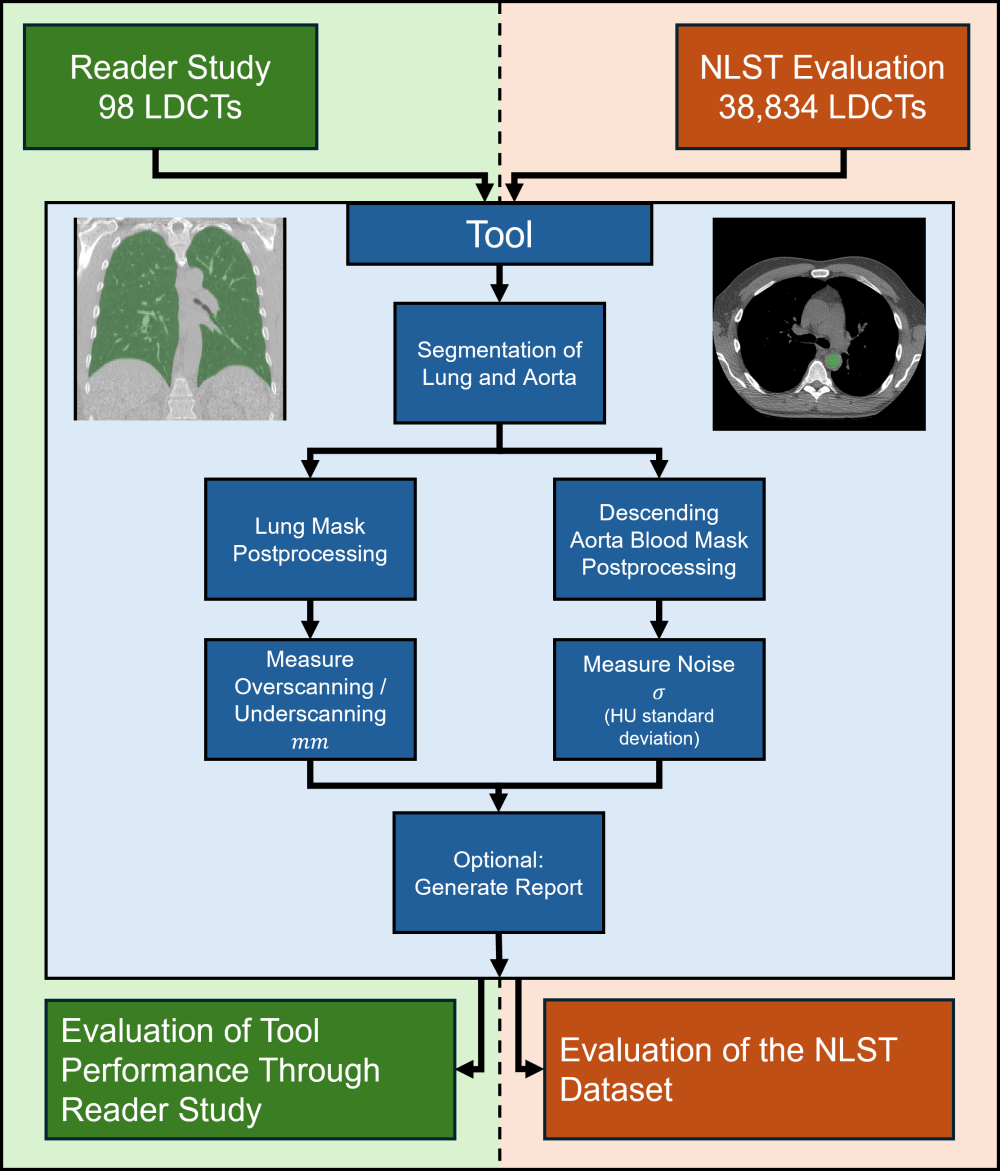
End-to-end workflow of the automated LDCT QA tool. Each LDCT volume is segmented (lungs and aorta), followed by post-processing to obtain a clean lung mask and a descending aortic-blood mask. Scan coverage is then quantified as cranial/caudal over- and underscanning (mm), and in-vivo noise is computed as the HU standard deviation within descending aortic blood. The pipeline can optionally generate a PDF report summarizing the results. Performance was assessed in a reader study (*N* = 98) and the method was applied at scale to 38,834 NLST LDCT scans.

To evaluate the tool’s efficacy, we conducted a reader study involving a radiologist with four years of experience, who analyzed 98 cases to assess both the quality of the segmentation masks and the diagnostic quality of the images. Furthermore, we evaluated a total of 38,834 LDCT scans to demonstrate the applicability of our tool to large datasets. An overview of the study and the tool is shown in Figure 1.

## 2. Related Work

The assessment of overscanning has been previously investigated by several researchers. Campbell et al. [14] reviewed 148 chest CTs, finding additional cranial slices in 97% and caudal slices in 98% of the scans. Further studies [15,16,17,18,19] also reviewed the issue of overscanning. A recent systematic review and meta-analysis concluded that AI-based models can reliably quantify manual overscanning in chest CT and consistently report substantially larger overscanning at the inferior than at the superior scan boundary [20]. Caudal overscanning is usually larger than cranial overscanning [16,18,19]. Moreover, the amount of overscanning can vary significantly, with some studies noting up to a threefold difference depending on the technologist performing the scan [18]. Systematic differences in the amount of overscanning can also be found between different hospitals [16]. Systematic overscanning can be reduced by raising awareness of the issue among clinical staff [19]. Beyond manual feedback interventions, published automated approaches have operationalized scan-range QA in different ways. Kim et al. proposed a deep learning–assisted overscan decision algorithm that mirrors KIAMI guideline landmark criteria [21], using landmark segmentation (thyroid cartilage as a substitute for the vocal cords and the kidney as inferior reference) combined with rule-based logic to determine over- and underscanning, and additionally estimating excess effective dose from the measured overscan length [11]. Colevray et al. quantified over-scanning by classifying axial slices into cervical, lung, and abdominal regions based on the first and last slices containing lung, enabling fast automated assessment at scale; for comparability with prior work, they also reported results after subtracting 2 cm margins in cranial and caudal directions [17]. These strategies rely on surrogate landmarks or slice-level region labeling, whereas our pipeline uses 3D organ segmentation to directly measure the cranio–caudal extent of lung parenchyma and explicitly flags underscanning when lung tissue reaches the scan boundary. In addition, our method extends scan-length QA by quantifying in-vivo image noise from descending aortic blood, providing complementary information on dose-related image quality.

In addition to overscanning, noise in CT scans is also integral to scan quality [22]. The noise in a CT scan is not homogeneous but varies across different regions [23]. In a laboratory environment, a phantom with known properties is commonly used to quantify noise [22,24]. To measure noise in vivo, it is common to annotate a homogeneous area in 2D or a homogeneous volume in 3D to use for the noise calculation. This has been applied to structures such as the aorta or liver tissue on 2D slices [25]. One approach is to use the 3D air volume in the trachea, since air is homogeneous [26]. Schuhbaeck et al. [27], however, used the blood in the aorta to measure noise. Air or blood are homogeneous on a scale captured by a CT scan. In an optimal CT, the number of photons hitting the detector is Poisson-distributed. Since the number of photons *N* is proportional to the amount of charge measured in mAs [13], and the noise σ corresponds to the standard deviation ($\sigma =\frac{1}{\sqrt{N}}$) of a Poisson distribution of photon counts *N* [13], the following theoretical relationship results:

(1) $$\sigma \propto \frac{1}{\sqrt{\mathrm{mAs}}}.$$

The amount of tube charge is given in mAs. Depending on the pitch of the CT, the amount of mAs a volume is exposed to changes. Therefore, effective mAs is defined in relation to pitch and mAs as follows [28]:

(2) $$\mathrm{effective}\;\mathrm{mAs}=\frac{\mathrm{mAs}}{\mathrm{pitch}}.$$

We make our tool and code publicly available: https://github.com/TruhnLab/ldct-quality-assurance, accessed on 4 November 2025.

## 3. Methods

For the lung and aorta segmentation, we employ TotalSegmentator (v2.4.0) [29], which is based on nn-Unet (v2.5.1) [30,31]. All analyses were performed in Python (v3.11.10). It segments each of the five lung lobes separately in the initial step, as well as the Aorta including its wall.

### 3.1. Over- and Underscanning

Each lung lobe mask is corrected by retaining only the largest connected component, as minor segmentation imperfections like single voxels are cut out by this approach. The segmentation masks of the five lung lobes are then combined into a single lung mask, which serves as the basis for determining underscanning or overscanning. Cranial or caudal underscanning is identified when the segmentation mask extends to the cranial-most or caudal-most slice, indicating that parts of the lungs may be missing from the scan. Overscanning is measured by calculating the number of slices cranial or caudal to the lung mask, multiplied by the slice thickness. In contrast to other studies [15,16,18], which define overscanning as anything beyond a 2 cm margin, we follow Campbell et al. [14], who define overscanning as anything extending beyond the lung parenchyma without any margin. We report this zero-margin distance as a continuous, maximally sensitive measure of excess scan range. Because small deviations are expected in practice (e.g., due to respiratory motion and uncertainty of the lung boundary on scouts), operational alerting is best performed using site-specific tolerance margins or data-driven thresholds applied to the measured distances.

### 3.2. Noise Calculation

Noise is commonly quantified using either the standard deviation or the signal-to-noise ratio (SNR). It is calculated by selecting a region or volume that should ideally exhibit homogeneous radiodensity. Previously, the liver or kidneys have been used for this purpose [25]. However, these tissues still have internal structures with varying radiodensities, which may introduce tissue-specific bias in the noise calculation. We therefore selected descending aortic blood as an in-vivo reference because it provides a large, approximately homogeneous volume that is consistently present in chest LDCT and can be segmented robustly; in contrast, tissue regions of interest (e.g., liver) are more heterogeneous and may introduce tissue-specific bias.

Following the approach of Schuhbaeck et al. [27], we used the blood in the aorta to measure noise. Since blood cells are microscopic and constantly in motion, blood appears homogeneous at the scale of CT imaging. Therefore, any inhomogeneity in the measured HU values of blood is attributable to factors such as quantum noise and other artifacts, rather than due to inhomogeneities in the measured medium. Since noise can vary across different CT image slices [23], and our metric should reflect the entire scan, we calculate the noise in a 3D volume of the aorta rather than on only a limited number of 2D slices.

To obtain voxels containing only blood, we begin with the segmentation mask of the Aorta provided by TotalSegmentator. As in previous steps, we retain only the largest connected component. The resulting segmentation includes the aortic wall and the aortic valve, neither of which are composed of blood. Additionally, using the entire aorta can disproportionately influence the noise metric because the aortic arch and ascending aorta occupy a larger volume in the upper slices, resulting in more voxel values from these regions and thus giving greater weight to the noise levels present in the top slices compared to the lower slices. Therefore, we applied post-processing to the aortic segmentation mask to isolate the descending aorta, yielding a more balanced sampling along the cranio–caudal axis and reducing cranial over-representation in the global noise estimate.

To isolate only the blood within the aorta, we apply binary erosion in the x-y plane to the segmentation mask, selecting the minimal number of voxels necessary to ensure a margin of at least 3.0 mm, since the aortic wall has an average thickness of 1.9 mm with a standard deviation of 0.3 mm [32]. This post-processing is designed to prioritize precision over recall: omitting some lumen voxels is acceptable for our purpose, whereas inclusion of non-blood structures (e.g., wall, valve, or calcifications) could substantially bias the HU distribution and thus the noise estimate. We further remove the ascending aorta by slicing through the segmentation mask from top to bottom until the segmentation volume splits into two components, one representing the ascending aorta and the other representing the descending aorta. As a result of this process, the aortic arch is partly cut away, the blood of the descending aorta is then selected as the dorsal connected component. The segmentation mask is then further trimmed to exclude regions below the lung, ensuring that only slices containing lung tissue are used for noise calculation.

Next, we compute the noise. We first calculate the mean $\mu_{\mathrm{raw}}$ and standard deviation $\sigma_{\mathrm{raw}}$ of all voxels covered by the mask.

Using the raw standard deviation presents a problem: it is dependent on image resolution. When an image is downscaled, voxels are combined, which reduces noise, since the image noise is theoretically inversely proportional to the square root of the number of photons detected [13]. Therefore, we normalize the noise by calculating how much of it would theoretically occur with a voxel size of $v_{\mathrm{norm}}=1\;{\mathrm{mm}}^{3}$, given the actual voxel volume $v_{\mathrm{raw}}$:

(3) $$\sigma_{\mathrm{norm}}=\sigma_{\mathrm{raw}}\sqrt{\frac{v_{\mathrm{raw}}}{v_{\mathrm{norm}}}}$$

Thus, the normalized noise standard deviation $\sigma_{\mathrm{norm}}$ serves as a voxel-size-independent metric.

### 3.3. Evaluation

**Figure 2:**
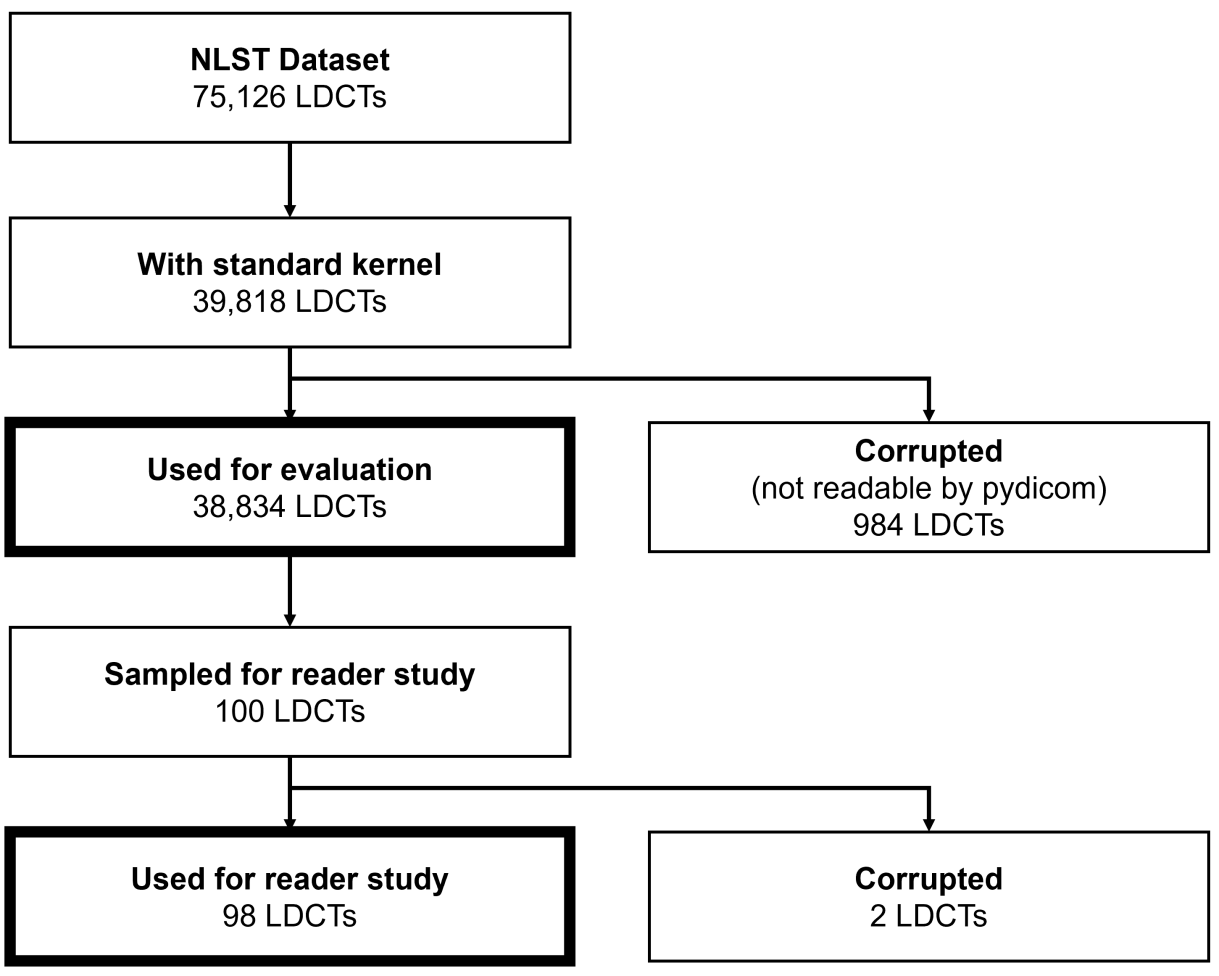
From the 75,126 NLST LDCT examinations, we restricted the cohort to scans reconstructed with a standard kernel (39,818) and excluded 984 corrupted/incomplete examinations (not readable with pydicom), resulting in 38,834 volumes for the main analysis. For the reader study, 100 volumes were sampled from this cohort; two corrupted scans were excluded, yielding 98 volumes for final evaluation.

To evaluate our approach, we rely on the NLST dataset [5,6], which contains LDCT scans obtained as part of lung cancer screenings. Figure 2 illustrates our exclusion process. The NLST dataset contains 75,126 LDCT screenings from 26,455 patients. For the evaluation of our tool, we conducted a reader study on 98 LDCT volumes. Furthermore, we assessed 38,834 LDCT volumes. We limited our evaluation to 39,818 volumes reconstructed using a standard kernel. This restriction ensured consistent noise measurements and reduced confounding from reconstruction-dependent noise characteristics. We further excluded 984 volumes for which the DICOM files were corrupted or incomplete in such a way that pydicom [33] was not able to generate a NIfTI file, leaving us with 38,834 usable volumes. These volumes originated from 14,218 patients, among whom 8203 were male and 6015 were female. Patient ages ranged from 55 to 75, with an average age of 61.4±5.0 (±standard deviation) at enrollment in the NLST study. Since the NLST study aimed to scan each patient at enrollment and then one and two years later, the patients’ ages during the scans varied accordingly. Patients had an average height of 172.2cm±9.7cm. For these volumes, we evaluated the amount of over- and underscanning, as well as the noise. For consortium-level subgroup analyses comparing the American College of Radiology Imaging Network (ACRIN) and the Lung Screening Study (LSS), we used two-sided independent-samples *t*-tests for continuous variables, Pearson’s chi-square tests for proportions, and Pearson correlation to assess associations with age.

To verify the performance of our method, we conducted a reader study in which a radiologist with four years of experience reviewed the processed lung segmentation masks and the segmentation masks of the blood in the descending aorta for our task. For lung mask evaluation, particular attention was paid to the accurate delineation of cranial and caudal boundaries to ensure complete coverage of lung tissue. For the aorta masks, the focus was on only having blood in the segmentation mask. It should not cover any part of the aortic wall, aortic valve, or other non-blood components, such as calcifications, since these could skew the noise calculation due to their different Hounsfield Unit (HU) values. Because our downstream tasks only require (i) correct cranio–caudal lung extent and (ii) a “pure” descending aortic-blood mask, we did not compute voxel-wise Dice similarity coefficients. Minor internal lung inaccuracies and undersegmentation of aortic blood are largely irrelevant, whereas false-positive voxels in the aorta (wall, valve, calcifications) would strongly bias the noise estimate; this asymmetry is not captured by the symmetric Dice metric. We therefore relied on a clinical, task-oriented visual assessment by an experienced radiologist, directly judging segmentation quality for coverage and aortic-lumen extraction. For the reader study, we sampled 100 volumes from the 38,834 available volumes. Two of these volumes had issues: one was incorrectly rotated, and the other contained corrupted incorrect HU values (<−19,000 HU). After excluding these, we ended up with 98 scans. These volumes originated from 97 different patients, of whom 62 were male and 35 were female. For the reader study sample, patient ages ranged from 55 to 74, with an average age of 61.41±5.49 at enrollment in the NLST study. Patients had an average height of 174.3cm±10.7cm. For the reader study, the radiologist evaluated the segmentation mask of the lung and the segmentation mask of the blood in the descending aorta on a scale from 1 (Insufficient) to 6 (Perfect) as described in Table 1. Additionally, the radiologist assessed the quality of the scans with respect to their diagnostic usability for detecting pulmonary nodules. The evaluation was done on the same scale from 1 to 6.

## 4. Results

### 4.1. Reader Study

The values in Table 2 indicate that the lung mask was rated Nearly Perfect or better in 90.8% of cases, while the mask for the descending aorta was rated Nearly Perfect or better in 96.9% of cases. When considering masks rated at least as “Good,” only one out of the 98 examined masks did not meet this quality standard. No segmentation mask was rated Poor or Insufficient.

**Figure 3:**
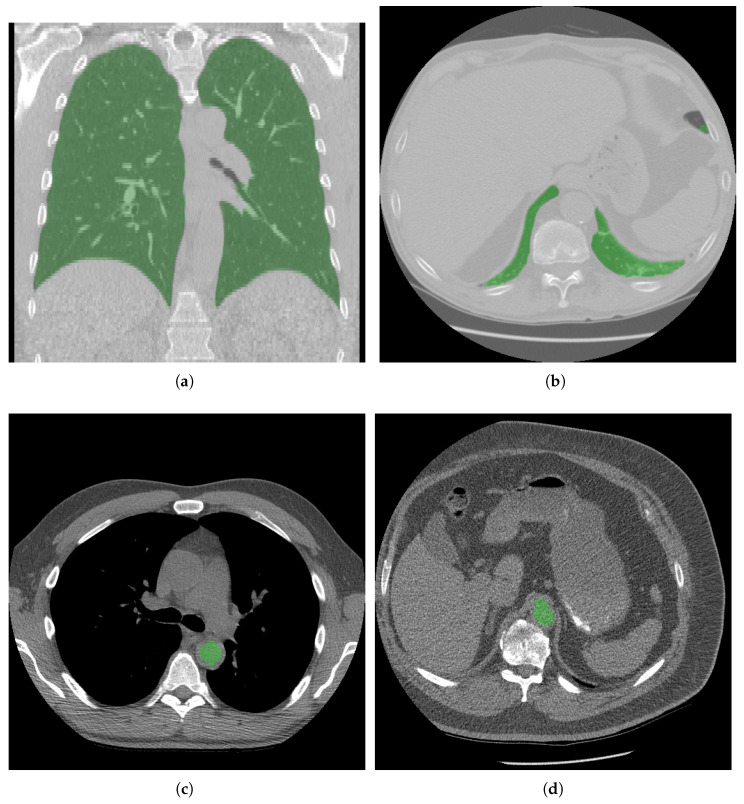
Examples from the reader study. Panels (**a**,**c**) show exemplary lung and aorta segmentations rated as “Perfect”. Panel (**b**) shows the lowest-rated lung segmentation (“Acceptable”): a small inferior portion of the lung is missing, but this did not affect the cranio–caudal extent measurement because the dorsal lung extended further caudally, correctly defining the lower boundary. Panel (**d**) shows an aortic-blood mask rated as “Good”: the segmentation slightly extends beyond the aortic lumen but does not include calcifications or adjacent structures and thus remains suitable for noise estimation.

Additionally, we calculated the Spearman correlation between the radiologist’s assessment of LDCT diagnostic usability and the measured volume-normalized noise $\sigma_{\mathrm{norm}}$. We observed a statistically significant (p=0.0051) but weak correlation of 0.281. Representative examples of the segmentation masks assessed in the reader study are shown in Figure 3.

### 4.2. NLST Evaluation

In addition to the reader study, we evaluated 38,834 LDCT volumes from the NLST dataset [5,6]. We measured both the occurrence of over- and underscanning as well as the noise levels. If overscanning occurred, the average overscanning distances were 31.21 mm with a standard deviation of 18.98 mm caudally and 14.54 mm with a standard deviation of 7.18 mm cranially.

Underscanning occurred caudally in 1694 cases, while it occurred cranially in 347 cases. This corresponds to 4.36% and 0.89% of the cases, respectively. Included in these are the 65 cases where both cranial and caudal underscanning occurred. In the remaining 36,858 cases, the lungs were fully captured, with at least one slice between the edge of the scan and the lungs.

For transparency and to facilitate comparison, Table 3 summarizes mean overscanning values reported in key studies alongside our results.

**Figure 4:**
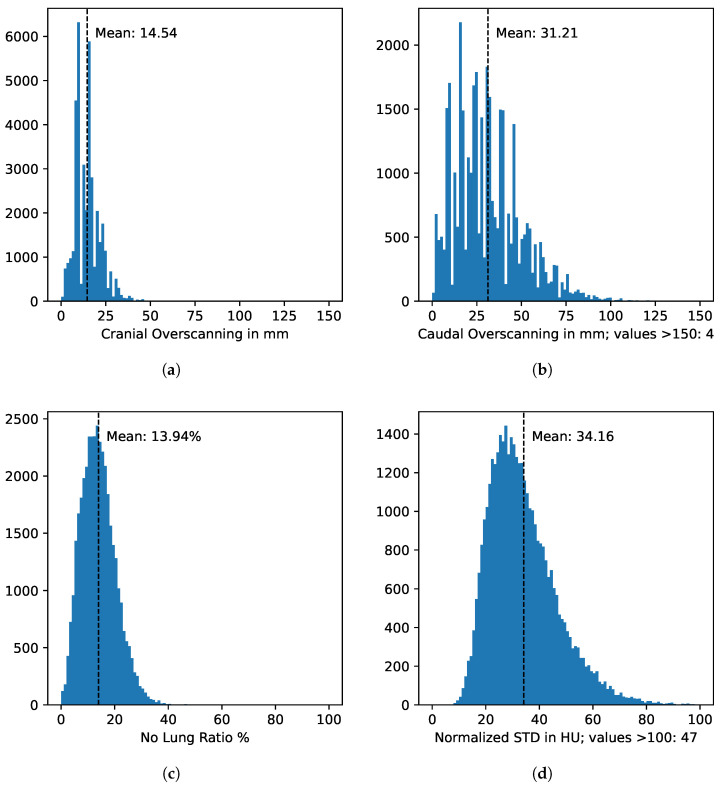
Caudal overscanning is more frequent and larger (**b**) than cranial overscanning (**a**). On average, 13.94% of slices in a scan contain no lung tissue (**c**). Panel (**d**) shows the distribution of normalized aortic-blood noise across LDCT scans.

**Figure 5:**
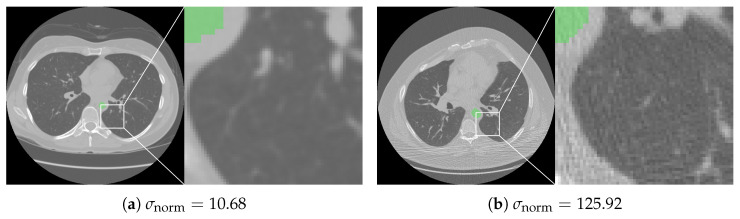
Two LDCT examples with very low and very high image noise. The example with higher $\sigma_{\mathrm{norm}}$ illustrates how increased noise appears as stronger graininess and mottling of the lung parenchyma.

The volume-normalized noise had a mean of 34.16 with a standard deviation of 22.51. The distribution is presented in Figure 4d. Figure 5 illustrates an example with high noise Figure 5b and an example with low noise Figure 5a. Center identifiers were not available in the publicly available NLST imaging/demographic files; therefore, we report consortium-level results for the two NLST screening consortia [5] (Table 4). Overscanning was significantly (p<0.001) larger in ACRIN than in LSS, whereas underscanning rates differed only slightly. Associations between age and total overscanning were negligible (ACRIN: r=0.013, p=0.10; LSS: r=0.031, p<0.001); among overscanned scans, age correlated weakly positively with caudal and weakly negatively with cranial overscanning (all |r|≤0.044), and males had slightly larger total overscanning than females in both consortia (both p<0.001).

## 5. Discussion

The reader study demonstrated that over 90.8% of the lung segmentation masks and more than 96.9% of the descending aorta blood masks were rated as “Nearly Perfect” or better. When considering masks rated at least as “Good,” only one out of the 98 examined masks did not meet this quality standard. These results indicate that our tool is suitable for fully automated quantification of overscanning and image noise to support clinical QA and targeted human review in LDCT scans. Additionally, if values are anomalous or unclear, a simple review of the segmentation masks can help identify or rule out possible errors. To ensure this, one only needs to verify that the lung segmentation mask correctly includes the lung and that the segmentation of the descending aorta’s blood contains only blood. For prospective clinical deployment, the tool could run as a background service receiving LDCT studies via standard DICOM routing and automatically returning QA outputs (metrics and a PDF report) to PACS, while forwarding key values and flags to the RIS to support protocol-specific alerts and rapid feedback for technologists and protocol owners. This supports exception-based prioritization by directing attention to studies with abnormal coverage or noise for targeted manual inspection, while routine cases can proceed without additional checks. This may reduce overall workload and associated costs, and can support dose optimization by identifying avoidable overscanning. A formal health-economic evaluation was beyond the scope of this study.

However, the low correlation of 0.281 between the subjective quality of a scan and the normalized noise indicates that image quality depends on more than just noise levels. The weak correlation between diagnostic usability and $\sigma_{\mathrm{norm}}$ is consistent with recent comprehensive reviews on CT image quality assessment, which emphasize that image noise is only one component of CT image quality and must be interpreted together with spatial resolution, contrast, and artifacts [36]. Other factors, such as beam-hardening artifacts, which do not impact the aorta, and patient movement, which reduces image quality, also play significant roles, even though they may not affect the measured noise in the aortic blood [37]. In other words, the radiologist’s diagnostic usability rating reflects a multi-factor assessment (e.g., sharpness/spatial resolution, contrast, and artifacts) rather than noise alone. Future work could therefore complement $\sigma_{\mathrm{norm}}$ with additional objective surrogates (e.g., sharpness/spatial-resolution measures, contrast-related metrics, and automated artifact indicators), although these quantities are often scanner-, protocol-, and task-dependent and do not admit a single universally accepted scalar “ground-truth” quality measure.

To demonstrate the applicability of our tool on large datasets, we evaluated 38,834 LDCT scans for overscanning, underscanning, and noise. We detected caudal underscanning in 1694 cases and cranial underscanning in 347 cases. On average, scans extended 31.21 mm ± 18.98 mm beyond the lung caudally and 14.54 mm ± 7.18 mm cranially, as shown in Figure 4a,b. The prevalence of greater caudal overscanning and the greater standard deviation compared to cranial overscanning has been reported in other studies [16,18,19], as well. Our findings that caudal overscanning is more pronounced than cranial overscanning are in line with a recent systematic review and meta-analysis of AI-based overscanning assessment in chest CT, which likewise reported markedly greater inferior than superior overscanning and emphasized the role of AI tools for protocol optimization and dose reduction [20]. One reason for this is that patients may move their diaphragm during the examination, leading to an axial shift of the caudal lung border, making its exact position unpredictable [38]. Additionally, the boundaries between air and soft tissue in the scout scan are not as easily identifiable as the boundaries of bones [38]. Accordingly, very small overranges may be unavoidable in routine practice; we therefore interpret overscanning as a continuous QA indicator and recommend center- and protocol-specific alert thresholds rather than a universal fixed margin.

The volume-normalized noise $\sigma_{\mathrm{norm}}$ had a mean of 34.16 with a standard deviation of 22.51. It can be observed that in extreme cases as shown in Figure 5, noise has a visibly significant impact on diagnostic quality. The most significant factor affecting image noise in CT scans is the slice thickness, with other influential factors including the reconstructed field-of-view, tube current (mAs), tube voltage (kVp), and lateral mis-centering [39].

### Limitations

One limitation of our study is that we used only a single CT reconstruction kernel. Reconstruction choices (e.g., kernel and iterative reconstruction) strongly influence measured noise and the dose–image-quality trade-off [40]. Therefore, absolute noise values (and any potential thresholds) are most comparable within the same reconstruction setting. Importantly, the core steps of our pipeline rely on organ segmentation with TotalSegmentator, which was trained on a large and heterogeneous dataset spanning multiple scanners and acquisition settings [29]; thus, we expect the segmentation-based coverage assessment to be robust across common kernels and protocols. Nevertheless, dedicated multi-center validation across additional kernels and iterative reconstruction techniques remains important future work. Furthermore, segmentation quality was assessed by a single radiologist; therefore, inter-reader agreement and repeatability of the visual ratings were not quantified. Multi-reader evaluation, including inter-rater agreement measures (e.g., weighted kappa), is an important next step to formally characterize reader-to-reader variability.

Additionally, the proposed method is retrospective in nature, i.e., it assesses overscanning, underscanning, and noise only after image acquisition has been completed; therefore, it does not reduce radiation dose during the acquisition process itself. Another limitation arises from potential misalignments in the LDCT volume, such as improper rotation during post-processing. This can cause errors in segmentation and impact the accuracy of over- and underscanning detection. To mitigate such failure modes in practice, automated integrity checks can be added prior to processing (e.g., verifying orientation consistency, slice spacing/thickness plausibility, and rescale metadata to avoid implausible HU ranges); studies failing these checks can be automatically flagged for re-orientation/re-conversion or excluded from analysis.

Finally, data curation imposes an additional limitation. In the 100-case reader-study sample, two scans were excluded (one with implausible HU values and one misoriented volume). We did not exhaustively curate all 38,834 NLST scans for acquisition or reconstruction failures (e.g., residual orientation errors, corrupted rescale metadata, slice-thickness inconsistencies, mis-centering, motion, or severe beam hardening), so a small fraction of undetected faulty scans may remain. Such errors are unlikely to bias the results toward unrealistically optimistic performance; if anything, they would tend to inflate overscanning estimates and noise measurements, making our results slightly conservative.

## 6. Conclusions

Our automated AI tool reliably assesses LDCT scan quality by measuring overscanning and image noise, supporting exception-based QA by reducing routine manual workload while preserving the need for targeted human review when abnormalities are flagged. In our study, lung segmentation masks were rated as “Nearly Perfect” or better in 90.8% of cases, demonstrating the tool’s effectiveness in accurately segmenting lung regions for quality assessment. The integration of such automated quality control measures can facilitate routine monitoring, enabling direct evaluation of quality assurance protocols and comparisons across different centers. By implementing our tool, institutions can improve the quality of LDCT scans both within individual centers and across multiple institutions. This enhancement leads to lower radiation doses through reduced over- and under-scanning and optimized noise levels, contributing to patient safety by minimizing radiation exposure risks. Ultimately, our tool supports more consistent and reliable LDCT screening practices, which are crucial for the early detection and treatment of lung cancer.

## Figures and Tables

**Table 1:** Rating scale from 1 to 6 with corresponding descriptions.

Rating	Description
6	Perfect
5	Nearly Perfect
4	Good
3	Acceptable
2	Poor
1	Insufficient

**Table 2:** Segmentation quality ratings for lung and aortic-lumen masks in the 98-scan reader study. The table shows how often (#) an LDCT scan was assigned a label. Additionally, the percentage indicates the proportion of masks that achieved at least that quality level, followed by the 95% confidence intervals.

	Lung Mask		Desc. Aorta Mask	
Label	#	Probability	#	Probability
Perfect	16	16.3% [CI: 9.6–25.2%]	69	70.4% [CI: 60.3–79.2%]
Nearly Perfect	73	90.8% [CI: 83.3–95.7%]	26	96.9% [CI: 91.3–99.4%]
Good	8	99.0% [CI: 94.4–100.0%]	3	100% [CI: 96.3–100.0%]
Acceptable	1	100% [CI: 96.3–100.0%]	0	100% [CI: 96.3–100.0%]
Poor	0	100% [CI: 96.3–100.0%]	0	100% [CI: 96.3–100.0%]
Insufficient	0	100% [CI: 96.3–100.0%]	0	100% [CI: 96.3–100.0%]

**Table 3:** Comparison of mean overscanning reported in prior AI studies and in our NLST evaluation. *N* denotes the number of examinations.

Study	*N*	Superior (mm)	Inferior (mm)	Overall (mm)
Colevray et al. [17]	1000	26.1	50.0	76.1
Huo et al. [34]	770	17.0	41.0	58.5
Kaviani et al. [35]	428	19.8±7.9	40.5±22.5	60.3
This work	38,834	14.54±7.18	31.21±18.98	45.75

**Table 4:** Consortium-stratified evaluation of scan coverage, normalized noise, and demographics in the NLST LDCT cohort: Total, American College of Radiology Imaging Network (ACRIN), and Lung Screening Study (LSS). *p*-values compare ACRIN vs. LSS and were computed using two-sided independent-samples *t*-tests for continuous variables and Pearson’s chi-square tests for proportions. Directional overscanning means are conditional; thus, mean total overscanning is not the sum of the directional means.

Metric	Total	ACRIN	LSS	*p*-Value
Sample size (*N*)	38,834	15,147	23,687	
Caudal overscanning (mm)	31.21±18.98	35.83±20.81	28.24±17.07	<0.001
Cranial overscanning (mm)	14.54±7.18	15.07±7.84	14.20±6.70	<0.001
Total overscanning (mm)	44.33±22.27	49.38±24.57	41.10±20.01	<0.001
Caudal underscanning (%)	4.36	4.07	4.55	0.028
Cranial underscanning (%)	0.89	1.18	0.71	<0.001
Normalized aorta noise (HU)	34.16±22.51	33.43±32.54	34.63±12.35	<0.001
Age (years)	61.36±4.99	61.48±5.00	61.28±4.97	<0.001
Male (%)	57.50	54.52	59.41	<0.001

## Data Availability

Our code is publicly available at https://github.com/TruhnLab/ldct-quality-assurance. The NLST dataset [5] used in this study is also available at https://www.cancerimagingarchive.net/collection/nlst, accessed on 30 July 2024.

## Abbreviations

The following abbreviations are used in this manuscript:

ACRIN	American College of Radiology Imaging Network
AI	Artificial Intelligence
CI	Confidence Interval
CNN	Convolutional Neural Network
CT	Computed Tomography
DICOM	Digital Imaging and Communications in Medicine
ERS	European Respiratory Society
ESR	European Society of Radiology
HU	Hounsfield Unit(s)
KIAMI	Korea Institute for Accreditation of Medical Imaging
kVp	Kilovoltage peak
LDCT	Low-Dose Computed Tomography
LSS	Lung Screening Study
Lung-RADS	Lung Imaging Reporting and Data System
mAs	Milliampere-seconds
NHS	National Health Service
NIfTI	Neuroimaging Informatics Technology Initiative
NLST	National Lung Screening Trial
PACS	Picture Archiving and Communication System
PDF	Portable Document Format
QA	Quality Assurance
RIS	Radiology Information System
SNR	Signal-to-Noise Ratio
